# Relationships among gut microbiota, plasma metabolites, and juvenile idiopathic arthritis: a mediation Mendelian randomization study

**DOI:** 10.3389/fmicb.2024.1363776

**Published:** 2024-03-28

**Authors:** Bingjun Gao, Zhe Wang, Kunyao Wang, Yinghan Lei, Yan Zhuang, Zhonghua Zhou, Junfei Chen

**Affiliations:** Department of Pediatric Surgery, Qilu Hospital of Shandong University, Jinan, China

**Keywords:** Mendelian randomization, gut microbiota, plasma metabolome, juvenile idiopathic arthritis, Bayesian weighted Mendelian randomization, mediation analysis

## Abstract

**Objective:**

The objective of this study is to investigate the causal relationship between gut microbiota and juvenile idiopathic arthritis, and to identify and quantify the potential role of plasma metabolites as mediators.

**Methods:**

Using summary-level data from genome-wide association studies, a two-sample Mendelian randomization was conducted involving 131 gut microbiota genus, 1,400 plasma metabolites, and juvenile idiopathic arthritis. Additionally, a two-step approach was employed to quantify the proportion of the effect of gut microbiota on juvenile idiopathic arthritis mediated by plasma metabolites. Effect estimation primarily utilized Inverse Variance Weighting, with further validation using Bayesian weighted Mendelian randomization.

**Results:**

In our MR analysis, a positive correlation was observed between *Rikenellaceae* and the risk of juvenile idiopathic arthritis, while *Dorea* showed a negative correlation with juvenile idiopathic arthritis risk. Mediation analysis indicated that Furaneol sulfate levels acted as a mediator between *Dorea* and juvenile idiopathic arthritis, with an indirect effect proportion of 19.94, 95% CI [8.86–31.03%].

**Conclusion:**

Our study confirms a causal relationship between specific microbial genus and juvenile idiopathic arthritis, and computes the proportion of the effect mediated by plasma metabolites, offering novel insights for clinical interventions in juvenile idiopathic arthritis.

## 1 Introduction

Juvenile Idiopathic Arthritis (JIA) is characterized by chronic arthritis, synovitis, and erosion of bone and cartilage. It is the most common rheumatic disease in young individuals, with an incidence ranging from 3.8 to 400 individuals per 100,000 (Costello et al., 2021). This condition not only damages the joints of affected children but often accompanies systemic involvement, such as macrophage activation syndrome (MAS), iridocyclitis, uveitis, and other multi-systemic manifestations (Arve-Butler et al., 2021). In untreated or severe cases, it can lead to deformities in hand joints or permanent eye damage, significantly impacting the physical and mental health of young individuals (Ravelli and Martini, 2007).

Over the course of evolutionary coexistence, the gut microbiota has formed a mutually dependent symbiotic relationship with the human body. The gut microbiota and its metabolic products play crucial roles in assisting digestion and absorption of food, synthesizing vitamins and energy, protecting the intestinal mucosal barrier, participating in essential metabolism, resisting invasion by pathogenic microbes, and regulating immune mechanisms (Brusca et al., 2014; Sathyabama et al., 2014). Alterations in the gut microbial community (dysbiosis) and decreased richness of the gut microbiota are emerging as relevant factors in the development of inflammatory and systemic autoimmune diseases (Lin et al., 2014; Collado et al., 2015; Rogers, 2015). Recent studies have indicated a potential connection between changes in the gut microbiota and JIA (Aggarwal et al., 2017; De Filippo et al., 2019; Öman et al., 2021; Chaudhary et al., 2023; Prinz et al., 2023; Shi et al., 2023).

In patients with enthesitis-related arthritis (ERA), an increase in the abundance of microbial genus such as *Rikenellaceae* and *Dorea* has been observed, while the abundance of *Prevotellaceae* is decreased (Aggarwal et al., 2017). Emmaline Prinz made a similar observation in mice, where an increase in *Rikenellaceae* altered the immune status of mice, making them more susceptible to arthritis (*R* = 0.43, *p* = 0.001) (Prinz et al., 2023). While animal models can provide valuable insights, they cannot substitute for clinical research. Currently, clinical studies on the relationship between gut microbiota and JIA are limited, and existing studies often suffer from small sample sizes. Observational studies are unable to eliminate confounding biases and reverse causation, and the pathophysiological mechanisms by which certain microbial genus operate can be partially compensated by dietary habits. Consequently, conflicting studies with opposing conclusions may arise. For instance, some studies suggest a decrease (Shi et al., 2023) or no difference (Öman et al., 2021) in *Rikenellaceae* and *Dorea* (De Filippo et al., 2019) in JIA, even though microbial differences may not impact the onset and progression of JIA (Chaudhary et al., 2023). Hence, a new approach is needed to clarify the causal relationship between gut microbiota and JIA.

The Mendelian Randomization (MR) study design is a robust tool in epidemiological research. Its core idea is to utilize genetic variations as instruments to assess the causal relationship between risk factors and specific diseases (Beeghly-Fadiel et al., 2020; Titova et al., 2020; Ahmed et al., 2021; Lu et al., 2021). Confounding factors are significant sources of interference in causal inference within epidemiological research. In MR studies, genetic variations follow the principles of mendelian inheritance, where alleles are randomly assigned to offspring, resembling the process of a randomized controlled trial (Davey Smith and Hemani, 2014; Davies et al., 2018). MR studies can effectively eliminate confounding factors and avoid reverse causation, which are challenges in observational studies (Hemani et al., 2018). Therefore, our aim is to employ MR to determine the causal relationship between gut microbiota and JIA, while also evaluating the extent to which plasma metabolites influence the association between gut microbiota and JIA.

## 2 Materials and methods

### 2.1 Study design

In this study, we conducted a two-sample MR analysis utilizing summary-level data from genome-wide association studies (GWAS) to assess the relationships between gut microbiota, plasma metabolites, and JIA. Sensitivity analyses were also performed to validate the robustness of the study results. MR relies on three fundamental assumptions: (Costello et al., 2021) the instrumental variable (IV) must exhibit a strong association with the exposure factor; (Arve-Butler et al., 2021) the IV should not be correlated with any confounding factors; (Ravelli and Martini, 2007) the IV can only influence the outcome variable through the exposure factor. These assumptions are integral to the effectiveness of Mendelian randomization and were rigorously tested in our study (Bandres-Ciga et al., 2020; Chen et al., 2020; Feng et al., 2020; Jones et al., 2020; Larsson et al., 2020; Saunders et al., 2020; Scheller Madrid et al., 2020; Zhu et al., 2020). This study is reported following the Strengthening the Reporting of Observational Studies in Epidemiology Using Mendelian Randomization guidelines (STROBE-MR, S1 Checklist).

### 2.2 Data source

The genetic variation data for gut microbiota were sourced from the largest meta-analysis of gut microbial composition genome-wide datasets conducted by the MiBioGen consortium to date (Kurilshikov et al., 2021). This study encompassed 18,340 individuals from 24 cohorts, predominantly of European ancestry (*n* = 13,266). Targeting the variable regions V4, V3-V4, and V1-V2 of the 16S rRNA gene, the analysis involved direct taxonomic classification to explore microbial composition. Microbial Quantitative Trait Loci (mbQTL) mapping analysis was performed to identify host genetic variations correlated with bacterial abundance levels in the gut microbiota. At the genus level, the lowest taxonomic classification in this study, 131 genera were identified, with an average abundance exceeding 1%, including 12 unknown genera. Consequently, 119 genus-level classification units were utilized in the current study for analysis (Li et al., 2022). The GWAS data for plasma metabolites and JIA were retrieved from the GWAS Catalog (GCST90199621-GCST90204603, GCST90010715), accessible at: http://ftp.Ebi.ac.uk/pub/databases/gwas/summary_statistics/. The plasma metabolite data comprised 1,091 blood metabolites and 309 metabolite ratios, involving 8,299 samples and approximately 150,000 SNP loci (Chen et al., 2023).

### 2.3 Instrument variables

To maximize the utility of instrumental variables (IVs), the following selection criteria were employed for the identification of potential IVs: (Costello et al., 2021) Single Nucleotide Polymorphisms (SNPs) within the locus showing significance below the threshold of *p* < 1.0 × 10^−5^ were chosen as potential IVs for each genus; (Arve-Butler et al., 2021) The 1,000 Genomes Project European sample data served as the reference panel for calculating Linkage Disequilibrium (LD) among SNPs. In regions where *r*^2^ < 0.001 (window size = 10,000 kb), only SNPs with the lowest *p* values and *F* > 10 were retained; (Ravelli and Martini, 2007) Allele frequency information was used to infer the forward strand allele when palindromic SNPs were present (Sanna et al., 2019; Li et al., 2022).

## 3 Mendelian randomization analysis

### 3.1 Primary analysis

We conducted a bidirectional two-sample MR to assess the causal relationship between gut microbiota and JIA. Various methods were employed to estimate MR effects, ensuring robustness. The Inverse Variance Weighting (IVW) method served as the primary approach, supplemented by Bayesian weighted Mendelian randomization (BWMR), MR-Egger, and Weighted Median methods, each tailored to different assumptions of instrument validity. The IVW method relies on the assumption that all SNPs are effective IVs, thus achieving accurate estimation results. On the other hand, BWMR considers the uncertainty caused by polygenicity leading to weak instrument effects and addresses violations of the IV assumption due to horizontal pleiotropy through Bayesian-weighted outlier detection (Zhao et al., 2020). MR-Egger assesses directional pleiotropy of IVs, with its intercept providing an estimate of the average pleiotropy of genetic variation. The Weighted Median method, compared to MR-Egger, exhibits higher precision and a smaller standard deviation.

### 3.2 Mediation mendelian randomization analysis

Furthermore, we employed a two-step MR design for mediation analysis (Yuan et al., 2022; Li Z. et al., 2024; Li F. et al., 2024) to investigate whether plasma metabolites mediate the pathway from gut microbiota to JIA. The overall effect can be decomposed into indirect effects and direct effects. The total impact of gut microbiota on JIA can be divided into (1) the direct impact of genus on JIA and (2) the indirect impact of genus on JIA mediated by plasma metabolites. We calculated the percentage of mediation effect by dividing the indirect effect by the total effect, simultaneously computing the 95% confidence interval.

### 3.3 Pleiotropy and heterogeneity analysis

Heterogeneity testing was performed using the MR Egger and IVW methods. Cochrane’s Q statistic was utilized to assess heterogeneity among genetic instruments, with *p* > 0.05 indicating no significant heterogeneity. The MR Egger regression equation was employed to evaluate horizontal pleiotropy of genetic instruments, with *p* > 0.05 suggesting the absence of horizontal pleiotropy. TwoSampleMR package in R software version 4.3.2 was utilized for allele harmonization and analysis. All statistical tests were two-sided, and statistical significance was considered at *p* < 0.05.

## 4 Results

### 4.1 The association between gut microbiota and JIA

In the MR analysis of gut microbiota and JIA, two genera were positively associated with the risk of JIA: *Rikenellaceae* (OR = 1.199, 95% CI [1.034–1.190], *p* = 0.015) and *Ruminococcus* (OR = 1.401, 95% CI [1.024–1.916], *p* = 0.034). Three genera showed a negative correlation with the risk of JIA: *Eubacteriumrectale* (OR = 0.722, 95% CI [0.530–0.983], *p* = 0.038), *Catenibacterium* (OR = 0.770, 95% CI [0.606–0.978], *p* = 0.032), and *Dorea* (OR = 0.669, 95% CI [0.489–0.915], *p* = 0.012). To validate these results, we used BWMR to calculate MR effects, confirming positive associations with the risk of JIA for *Rikenellaceae* (OR = 1.184, 95% CI [1.024–1.368], *p* = 0.022) and *Ruminococcus* (OR = 1.457, 95% CI [1.049–2.022], *p* = 0.024); and negative associations for *Eubacteriumrectale* (OR = 0.709, 95% CI [0.509–0.988], *p* = 0.042), *Catenibacterium* (OR = 0.757, 95% CI [0.586–0.976], *p* = 0.032), and *Dorea* (OR = 0.647, 95% CI [0.456–0.917], *p* = 0.014) reducing the risk of JIA.

Results from reverse MR analysis indicated that JIA can influence the abundance of *Eubacteriumrectale* (OR = 0.989, 95% CI [0.981–0.997], *p* = 0.011), *Catenibacterium* (OR = 0.976, 95% CI [0.955–0.998], *p* = 0.039), and *Ruminococcus* (OR = 1.014, 95% CI [1.005–1.023], *p* = 0.001). In BWMR, *Eubacteriumrectale* (OR = 0.989, 95% CI [0.980–0.997], *p* = 0.012), *Catenibacterium* (OR = 0.976, 95% CI [0.954–0.999], *p* = 0.041), *Ruminococcus* (OR = 1.015, 95% CI [1.006–1.024], *p* = 0.001), *Rikenellaceae* (*p* = 0.207), and *Dorea* (*p* = 0.136) demonstrated a unidirectional causal relationship. Thus, the genus with a unidirectional causal relationship were *Rikenellaceae* and *Dorea*.

### 4.2 The association between plasma metabolites and JIA

There are a total of 55 metabolites associated with changes in the risk of JIA (Supplementary Table 18). Metabolites that decrease the risk of JIA include Cysteine-glutathione disulfide levels (OR = 0.802, 95% CI [0.679–0.946], *p* = 0.009) and 2,6-dihydroxybenzoic acid levels (OR = 0.774, 95% CI [0.647–0.925], *p* = 0.005). Metabolites that increase the risk of JIA include 1-stearoyl-2-oleoyl levels (OR = 1.167, 95% CI [1.008–1.350], *p* = 0.038) and Furaneol sulfate levels (OR = 1.428, 95% CI [1.155–1.766], *p* = 0.001). To further validate these results, BWMR was used to calculate effect values for 1-stearoyl-2-oleoyl levels (OR = 1.173, 95% CI [1.003–1.372], *p* = 0.046), Cysteine-glutathione disulfide levels (OR = 0.798, 95% CI [0.671–0.950], *p* = 0.012), Furaneol sulfate levels (OR = 1.361, 95% CI [1.0.73–1.725], *p* = 0.011), and 2,6-dihydroxybenzoic acid levels (OR = 0.764, 95% CI [0.638–0.915], *p* = 0.003).

### 4.3 The association between gut microbiota and plasma metabolites

*Rikenellaceae* decreases 1-stearoyl-2-oleoyl levels (OR = 0.902, 95% CI [0.836–0.972], *p* = 0.006). *Dorea* reduces Furaneol sulfate levels (OR = 0.784, 95% CI [0.621–0.989], *p* = 0.040). To further validate these results, effect values were calculated using BWMR, indicating that *Rikenellaceae* decreases 1-stearoyl-2-oleoyl levels (OR = 0.895, 95% CI [0.830–0.967], *p* = 0.004), and *Dorea* reduces Furaneol sulfate levels (OR = 0.778, 95% CI [0.626–0.967], *p* = 0.002).

**Figure 1:**
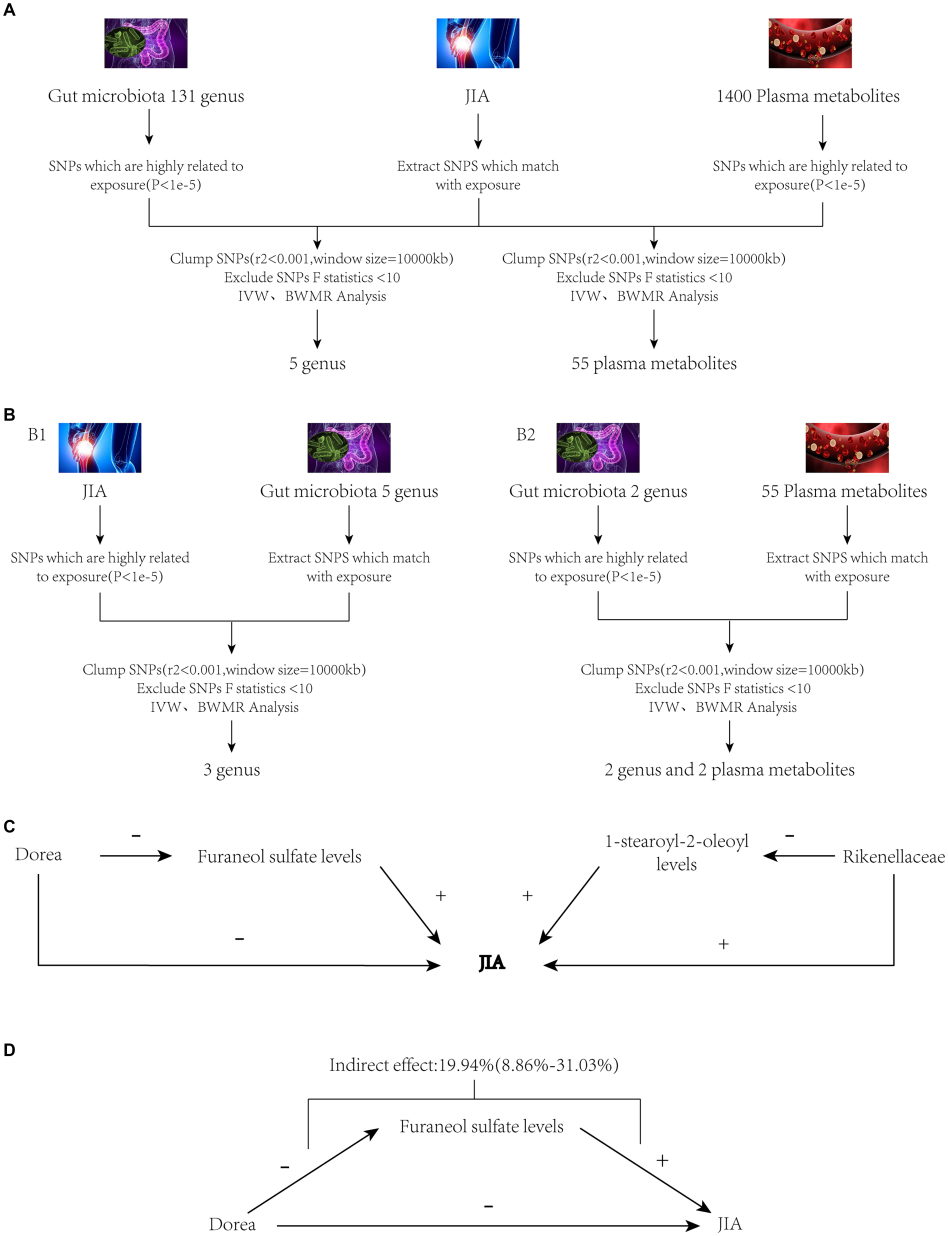
Study workflow. Panel **(A)**: JIA as the outcome variable, with gut microbiota and plasma metabolites as exposures. Panel **(B1)**: JIA as the exposure variable, with gut microbiota as the outcome variable. Panel **(B2)**: Gut microbiota as the exposure variable, with plasma metabolites as the outcome variable. Panel **(C)**: Preliminary mediation analysis obtained after MR screening as described above. Panel **(D)**: Final mediation MR analysis obtained after thorough screening. JIA, Juvenile Idiopathic Arthritis; MR, Mendelian Randomization; SNPs, Single nucleotide polymorphisms; BWMR, Bayesian weighted Mendelian randomization; IVW, Inverse Variance Weighting.

In the two-step mediation analysis, the indirect effect of *Rikenellaceae* on JIA through 1-stearoyl-2-oleoyl levels was opposite in direction to the total effect of *Rikenellaceae* on JIA. Therefore, 1-stearoyl-2-oleoyl levels cannot serve as a mediator for the relationship between *Rikenellaceae* and JIA. Conversely, *Dorea*’s indirect effect on JIA through Furaneol sulfate levels was in the same direction as the total effect of *Dorea* on JIA and demonstrated causality under both IVW and BWMR verification. Thus, Furaneol sulfate levels can be considered a mediator for the relationship between *Dorea* and JIA, with a mediation effect proportion of 19.94% (95% CI [8.86–31.03%]). The analytical workflow is depicted in Figure 1.

**Figure 2:**
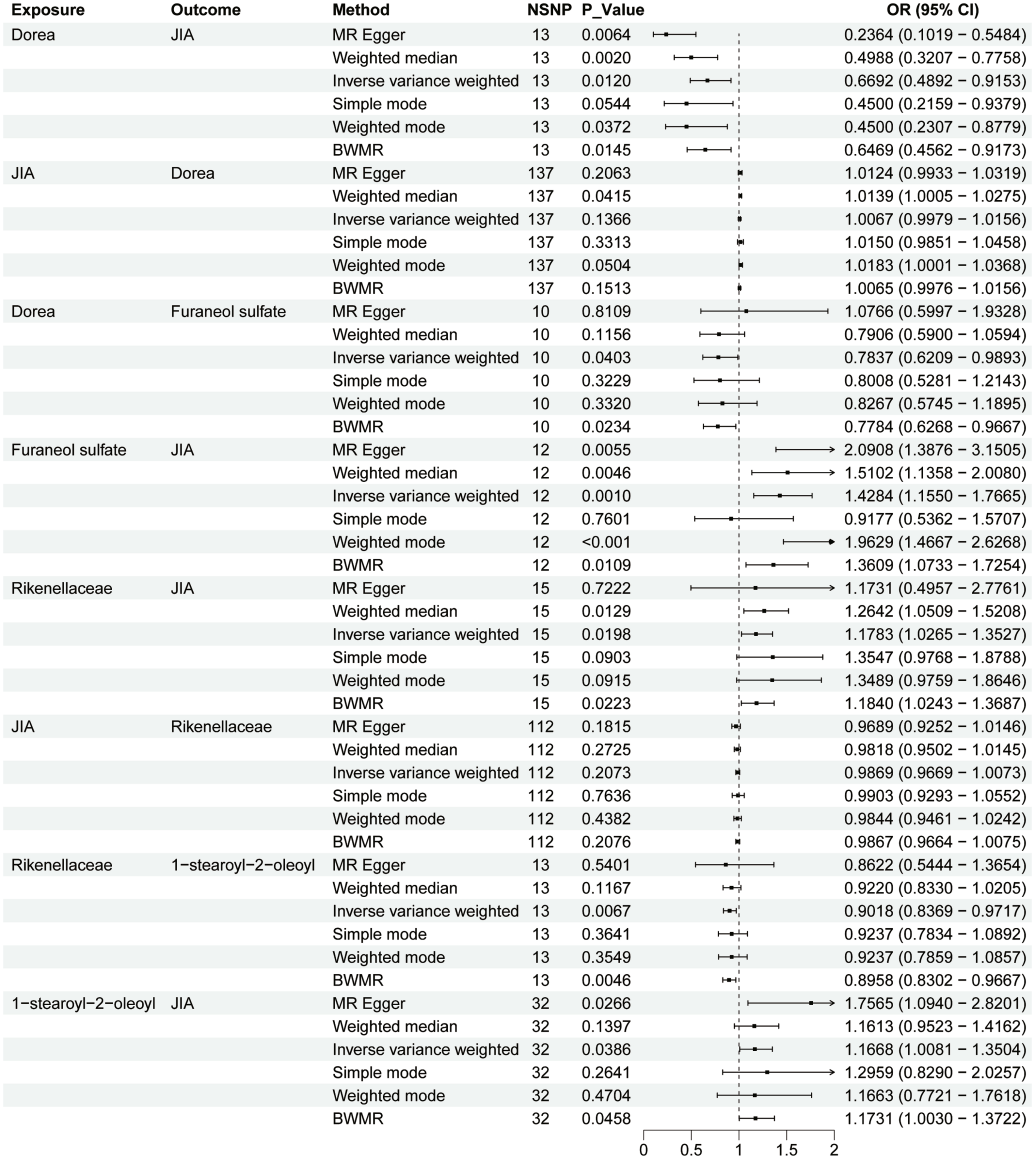
Forest plot to visualize the causal effects of plasma metabolites with gut microbiota and juvenile idiopathic arthritis.

**Figure 3:**
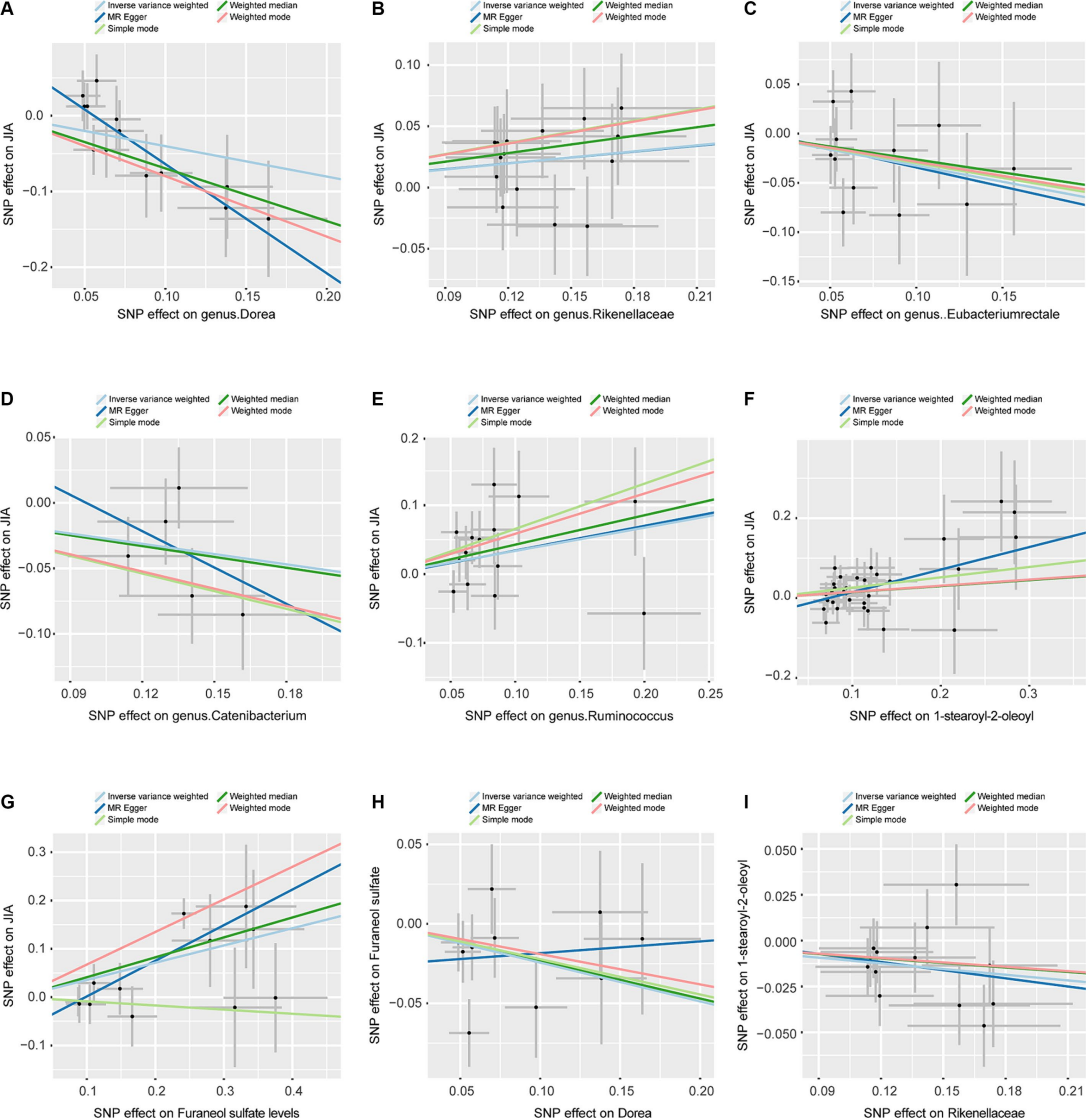
Scatterplots, the horizontal axis represents the SNP effect on exposure, while the vertical axis illustrates the SNP effect on the outcome. **(A)** Represents the MR between Dorea and JIA. **(B)** Represents the MR between Rikenellaceae and JIA. **(C)** Represents the MR between Eubacteriumrectale and JIA. **(D)** Represents the MR between Catenibacterium and JIA. **(E)** Represents the MR between Ruminococcus and JIA. **(F)** Represents the MR between 1-stearoyl-2-oleoyl and JIA. **(G)** Represents the MR between Furaneol sulfate and JIA. **(H)** Represents the MR between Dorea and Furaneol sulfate. **(I)** Represents the MR between Rikenellaceae and 1-stearoyl-2-oleoyl. JIA, Juvenile Idiopathic Arthritis; MR, Mendelian Randomization; SNP, Single nucleotide polymorphism.

**Figure 4:**
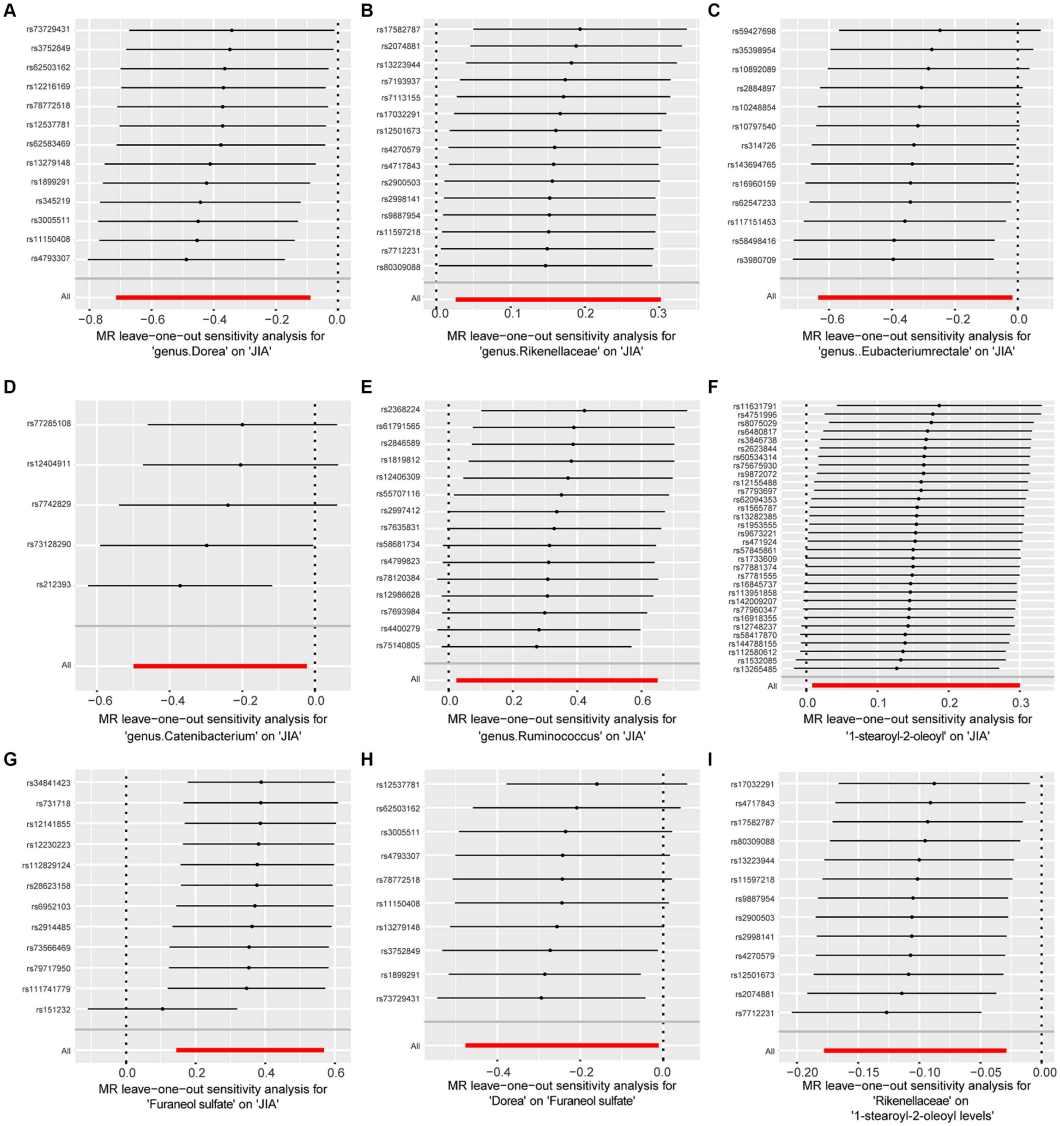
Forest plot to visualize the impact of removing a single SNP on the overall effect. **(A)** Represents the MR between Dorea and JIA. **(B)** Represents the MR between Rikenellaceae and JIA. **(C)** Represents the MR between Eubacteriumrectale and JIA. **(D)** Represents the MR between Catenibacterium and JIA. **(E)** Represents the MR between Ruminococcus and JIA. **(F)** Represents the MR between 1-stearoyl-2-oleoyl and JIA. **(G)** Represents the MR between Furaneol sulfate and JIA. **(H)** Represents the MR between Dorea and Furaneol sulfate. **(I)** Represents the MR between Rikenellaceae and 1-stearoyl-2-oleoyl. JIA, Juvenile Idiopathic Arthritis; MR, Mendelian Randomization; SNP, Single nucleotide polymorphism.

To assess the stability of these results, Mr-Egger and Mr-PRESSO tests were conducted on the included SNP loci. Neither test revealed potential horizontal pleiotropy (*p* > 0.05), and the funnel plot did not indicate bias in the study. The corrected Cochran’s Q statistic showed no significant heterogeneity in the effects of the included SNPs (*p* > 0.90). Additionally, a leave-one-out sensitivity analysis was performed to evaluate the influence of each SNP locus on the overall causal relationship. When systematically removing individual SNPs and reanalyzing, the results showed no significant differences in the observed causal relationship, emphasizing that the estimated effects cannot be attributed to any single genetic tool. All MR Analysis results are shown in the Figure 2. The results of heterogeneity test and horizontal pleiotropy are in the Supplementary files. The relationship between exposure and outcome is presented in Figure 3 in the form of a scatter plot. The robustness of the results is assessed using the leave-one-out method, as depicted in Figure 4.

## 5 Discussion

In this large-scale MR analysis, we identified causal relationships between 5 genus and JIA, with *Rikenellaceae* and *Dorea* demonstrating unidirectional causality. *Rikenellaceae* showed a positive correlation with the risk of JIA. Mediation analysis indicated that Furaneol sulfate levels mediate the effect of *Dorea* on JIA by 19.94% (95% CI [8.86–31.03%]). This analysis underscores the connection between gut microbiota and JIA, emphasizing the mediating role of Furaneol sulfate levels (Figures 3, 4).

Gut microbiota regulates immune responses through two main mechanisms: direct recognition by immune cells and metabolite-mediated immune response modulation. Short-chain fatty acids (SCFAs) not only regulate immune responses in the gut but also impact systemic multi-system functions. SCFAs act as inhibitors of histone deacetylases (HDACs) and ligands for G protein-coupled receptors (GPCRs), serving as signaling molecules that influence the immune system. SCFAs (butyrate, propionate, and acetate) as HDAC inhibitors affect peripheral blood mononuclear cells, leading to the inactivation of NF-κB and reduced production of the pro-inflammatory cytokine TNF-α (Usami et al., 2008). Another study further demonstrated that butyrate can regulate the function of intestinal macrophages by inhibiting HDAC, downregulating the production of pro-inflammatory factors, including NO, IL-6, and IL-12 (Chang et al., 2014). Bacteria such as *Faecalibacterium*, *Ruminococcaceae*, *Parabacteroides*, *Clostridiales vadin BB60 group*, and *Roseburia* are known producers of butyrate (Louis and Flint, 2009; Louis and Flint, 2017). *Parabacteroides* and *Clostridiales vadin BB60 group* also produce other SCFAs such as acetate, propionate, and hexanoate (Che et al., 2019; Lei et al., 2021). Our results suggest that the protective effects of *Eubacteriumrectale*, *Catenibacterium*, and *Dorea* against JIA may be attributed to their role in SCFA production. Studies have indicated a close association between *Dorea* and autoimmune diseases (Wang et al., 2023), with involvement in regulating the body’s immune checkpoint inhibition response (Liu et al., 2023), suggesting a potential link between *Dorea* and JIA.

Observational studies generally suggest a close association between gut microbiota and autoimmune diseases. In a comparison of baseline samples from Italian patients and healthy controls, patients exhibited an increase in the abundance of *Erysipelotrichaceae*, *Faecalibacterium prausnitzii*, *Fusobacterium*, *Enterococcus*, and *Ruminococcaceae*, while *Allobaculum*, *Gemellaceae*, *Propionibacterium acnes*, and *Turicibacter* were less abundant compared to healthy controls (van Dijkhuizen et al., 2019). Although increasing evidence indicates the role of gut microbiota dysbiosis in JIA, this field is still in its early stages. To date, studies on the composition and changes in the gut microbiota of JIA children, compared to healthy subjects, have been descriptive, and the potential functions of the microbiota remain speculative, making it challenging to establish causal relationships between microbial changes and JIA (De Filippo et al., 2019). Recent research suggests that metabolic processes and metabolites can influence disease risk and provide therapeutic targets. Understanding the causal role of metabolites in disease etiology can offer actionable intervention points for treatment. One approach to assessing the role of metabolites in disease outcomes is through human genetics. Many metabolite levels have a high heritability, providing an opportunity for MR, a causal inference method that uses genetic variation as IVs to test the role of exposures in disease outcomes. As alleles are randomly assigned at conception, this randomization process often breaks the confounding with most risk factors, thereby reducing the tendency to confound results (Chen et al., 2023). Interestingly, research has found that dysbiosis of the gut microbiota can influence autoimmunity (Bellés et al., 2022) and the progression (Chung and Kasper, 2010; Stoll et al., 2014) of inflammatory diseases by altering metabolite levels and ratios (Chen et al., 2023). To our knowledge, there is currently no in-depth study on the relationship between plasma metabolites and JIA. Our study results suggest that Furaneol sulfate levels may bridge the causal relationship between gut microbiota and JIA, laying the groundwork for exploring the intersection of gut microbiota and JIA and potentially inspiring new strategies for JIA treatment.

It is noteworthy that research results on the differences in gut flora in JIA patients are inconsistent. In a study of Italian children, the gut microbial diversity in JIA patients was significantly reduced compared to healthy subjects, with an increased abundance of the *Dorea* genus in JIA patients (De Filippo et al., 2019). The microbial composition of JIA children did not show significant differences from their healthy siblings (Öman et al., 2021). Reasons for result heterogeneity include, firstly, studies may not have considered potential confounding factors such as gender, race, diet, delivery mode, and medication use (Xin et al., 2021). Overall, JIA is more common in girls than boys (ratio of 2: 1). Systemic juvenile idiopathic arthritis is believed to occur in both sexes, while enthesitis-related arthritis is more common in boys than girls (Thierry et al., 2014). Different regions and races have different dietary habits; a high-fiber diet increases the ratio of *Bacillota*/*Bacteroidota* in the gut, promoting SCFA production, while a low-fiber, high-protein diet increases pro-inflammatory cytokine levels like IL-2 and IL-6 (De Filippo et al., 2010, 2019). Secondly, bacterial classification may differ between studies, contributing to result heterogeneity.

In systemic juvenile idiopathic arthritis (sJIA) patients, elevated levels of IL-6 have been found in blood and synovial fluid, and they are associated with disease activity (De Benedetti et al., 1997). IL-6 antagonists have been shown to be a potential therapy for refractory inflammatory diseases, similar to traditional corticosteroids (Ataie-Kachoie et al., 2013). In mice, copper disrupts the ecological balance and diversity of the gut microbiota, increasing *Enterobacteriaceae* while reducing the abundance of *Bacteroidaceae*, *Ruminococcaceae*, and *Lachnospiraceae*. However, *Bacillus subtilis* reverses copper toxicity by increasing taurine and L-glutamate levels while decreasing phosphatidylcholine and phosphatidylethanolamine, moving toward alleviating metabolic disruption (Gao et al., 2023). Olive oil supplementation prevents type 1 diabetes in NOD mice by modulating the gut microbiota and serum metabolites (Wang et al., 2023). Therefore, we hypothesize that finding suitable drugs or targets to reduce Furaneol sulfate levels may slow down the progression of JIA.

We were the first to employ MR to investigate the causal relationships among gut microbiota, plasma metabolites, and JIA. We not only utilized various common sensitivity analyses but also mitigated the impacts of confounding factors and reverse causation. Our preliminary findings suggest a causal relationship between gut microbiota and JIA, as well as the intermediary factors. This provides further theoretical support for the treatment and prevention of JIA and introduces new approaches to its management. For instance, JIA could initially be controlled through the regulation of specific gut microbiota via diet, medications, or other means. Additionally, co-regulating plasma metabolite levels may lead to significant breakthroughs in JIA prevention and treatment.

However, our study has several limitations. Firstly, the analyzed population is predominantly of European descent, somewhat limiting the generalizability of the findings. Secondly, due to limited GWAS data, we did not explore each subtype of JIA individually. Thirdly, our results remain theoretical and have not been validated through clinical or animal experiments, leaving the specific mechanisms unclear. Further cellular and animal experiments are needed to elucidate these mechanisms. Subsequently, we will seek to confirm the reliability of the results at the population level through randomized clinical trials. Lastly, we observed that only 19.94% of the effect was mediated by plasma metabolite Furaneol sulfate levels, which is relatively low, necessitating more research to quantify other mediators.

## 6 Conclusion

Our mediation analysis using MR indicates a causal relationship among gut microbiota, plasma metabolites, and JIA. Specifically, the metabolic pathway involving Furaneol sulfate mediates the regulatory effect of *Dorea* on JIA. The genetic evidence provided by our study underscores the connections between gut microbiota, plasma metabolites, and JIA. This suggests that future interventions could focus on improving gut microbiota and co-regulating Furaneol sulfate levels through medications, thereby enhancing prevention and treatment strategies for JIA.

## Data availability statement

The datasets presented in this study can be found in online repositories. The names of the repository/repositories and accession number(s) can be found in the article/Supplementary material.

## Ethics statement

Ethical approval was not required for the studies involving humans because this two-sample MR study is based on publicly available summary data from genome-wide association studies (GWAS). All of these studies have obtained approval from the relevant institutional review boards, and participants have provided informed consent. The studies were conducted in accordance with the local legislation and institutional requirements. Written informed consent for participation was not required from the participants or the participants’ legal guardians/next of kin in accordance with the national legislation and institutional requirements.

## Author contributions

BG: Conceptualization, Data curation, Formal analysis, Funding acquisition, Investigation, Methodology, Project administration, Resources, Software, Supervision, Validation, Visualization, Writing – original draft, Writing – review & editing. ZW: Writing – original draft, Writing – review & editing, Conceptualization, Data curation, Investigation, Methodology, Software, Supervision. KW: Conceptualization, Data curation, Investigation, Methodology, Software, Supervision, Writing – original draft, Writing – review & editing. YL: Formal analysis, Funding acquisition, Project administration, Resources, Validation, Visualization, Writing – original draft, Writing – review & editing. YZ: Conceptualization, Formal analysis, Funding acquisition, Investigation, Project administration, Resources, Software, Supervision, Validation, Visualization, Writing – original draft, Writing – review & editing. ZZ: Data curation, Formal analysis, Funding acquisition, Methodology, Project administration, Resources, Supervision, Validation, Visualization, Writing – original draft, Writing – review & editing. JC: Conceptualization, Data curation, Formal analysis, Funding acquisition, Investigation, Methodology, Project administration, Resources, Software, Supervision, Validation, Visualization, Writing – original draft, Writing – review & editing.
