# Supplementary material for: Relationships among gut microbiota, plasma metabolites, and juvenile idiopathic arthritis: a mediation Mendelian randomization study
Source: Front Microbiol. 2024 Mar 28;15:1363776. doi: 10.3389/fmicb.2024.1363776 (PMC11007183; doi:10.3389/fmicb.2024.1363776)
Supplement: Supplementary file 2 [file Data_Sheet_1.PDF]

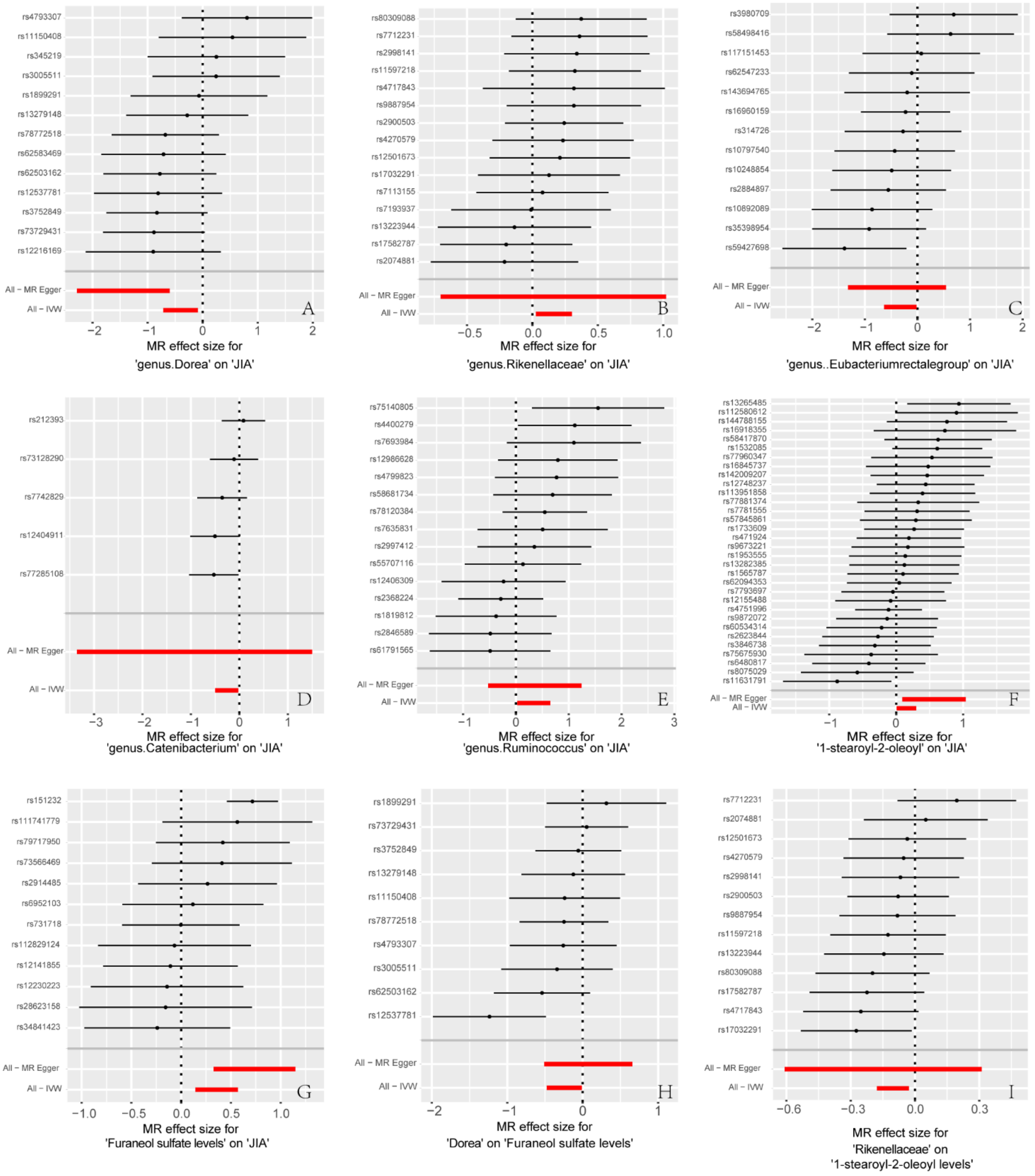


**Supplementary Figure 1** forest plot that reflects causality

A represents the causal relationship between Dorea and JIA;B represents the causal relationship between Rikenellaceae and JIA;C represents the causal relationship between Eubacteriumrectale and JIA;D represents the causal relationship between Catenibacterium and JIA; E represents the causal relationship between Ruminococcus and JIA; F represents the causal relationship between 1-stearoyl-2-oleoyl and JIA;G represents the causal relationship between Furaneol sulfate and JIA; H represents the causal relationship between Dorea and Furaneol sulfate; I represents the causal relationship between Rikenellaceae and 1-stearoyl-2-oleoyl.

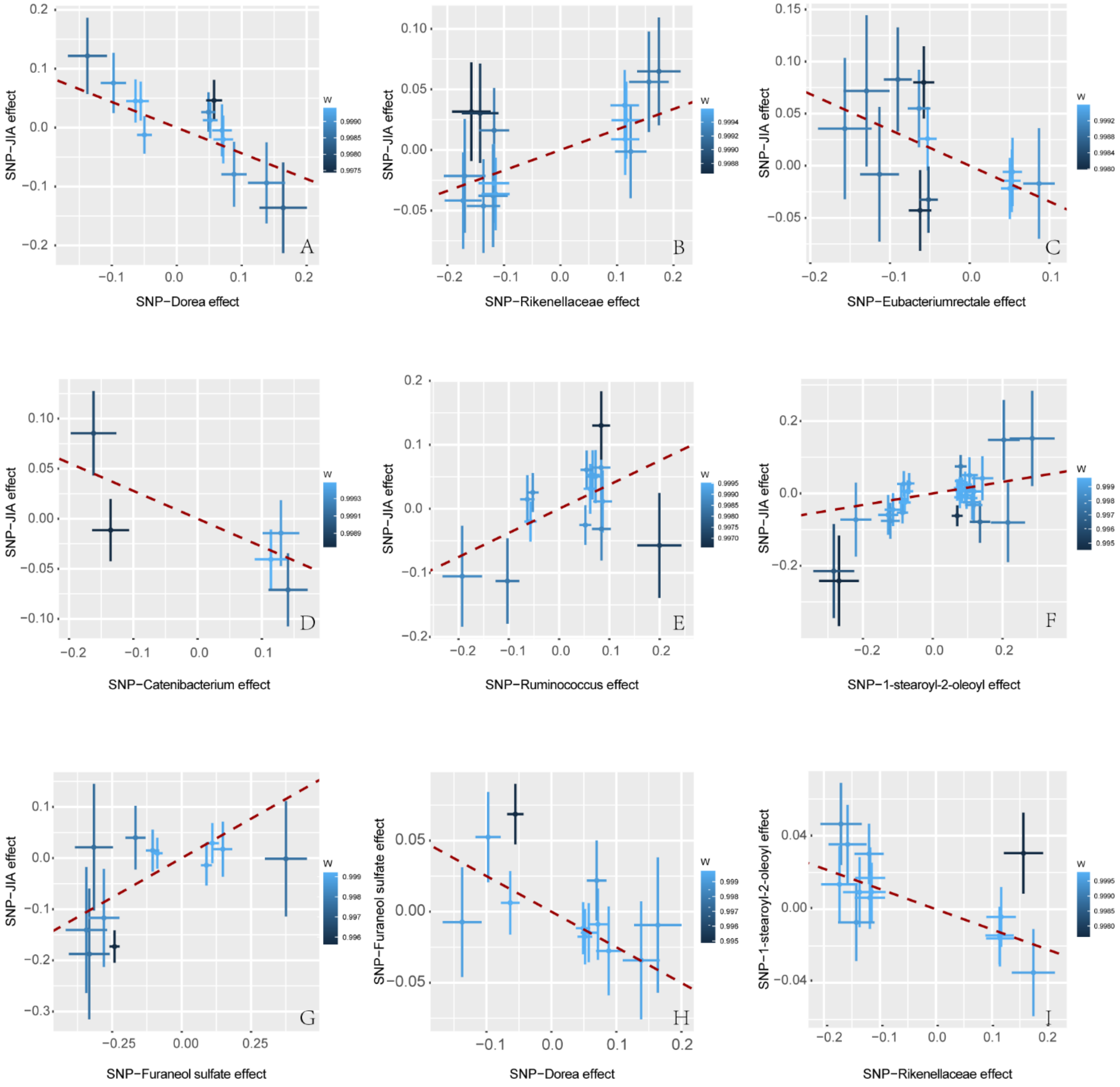


**Supplementary Figure 2** Plot of weighted data and its regression result

A represents the causal relationship between Dorea and JIA;B represents the causal relationship between Rikenellaceae and JIA;C represents the causal relationship between Eubacteriumrectale and JIA;D represents the causal relationship between Catenibacterium and JIA; E represents the causal relationship between Ruminococcus and JIA; F represents the causal relationship between 1-stearoyl-2-oleoyl and JIA;G represents the causal relationship between Furaneol sulfate and JIA; H represents the causal relationship between Dorea and Furaneol sulfate; I represents the causal relationship between Rikenellaceae and 1-stearoyl-2-oleoyl.

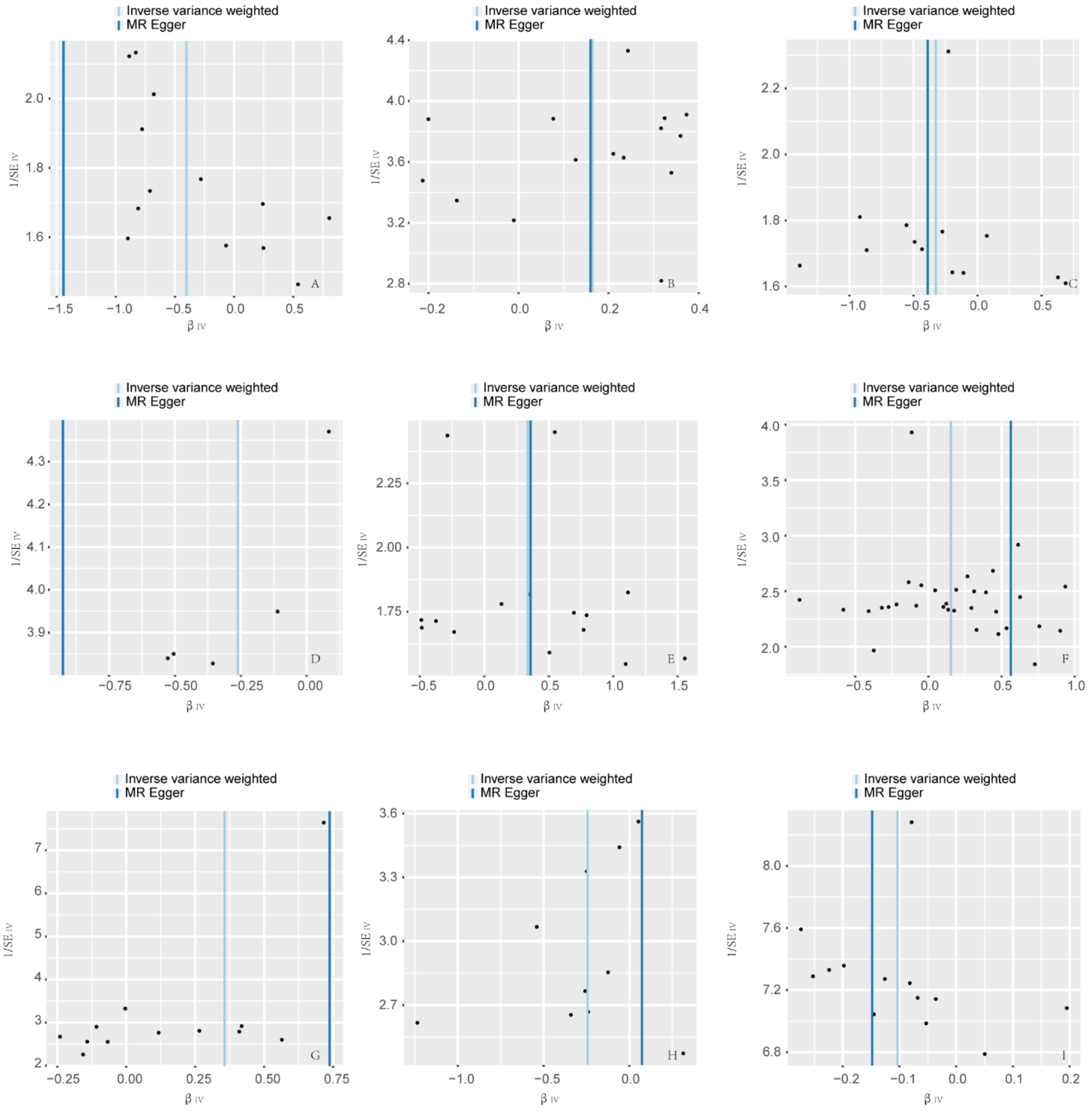


**Supplementary Figure 3** funnel plot of MR analysis

A represents the causal relationship between Dorea and JIA;B represents the causal relationship between Rikenellaceae and JIA;C represents the causal relationship between Eubacteriumrectale and JIA;D represents the causal relationship between Catenibacterium and JIA; E represents the causal relationship between Ruminococcus and JIA; F represents the causal relationship between 1-stearoyl-2-oleoyl and JIA;G represents the causal relationship between Furaneol sulfate and JIA; H represents the causal relationship between Dorea and Furaneol sulfate; I represents the causal relationship between Rikenellaceae and 1-stearoyl-2-oleoyl.

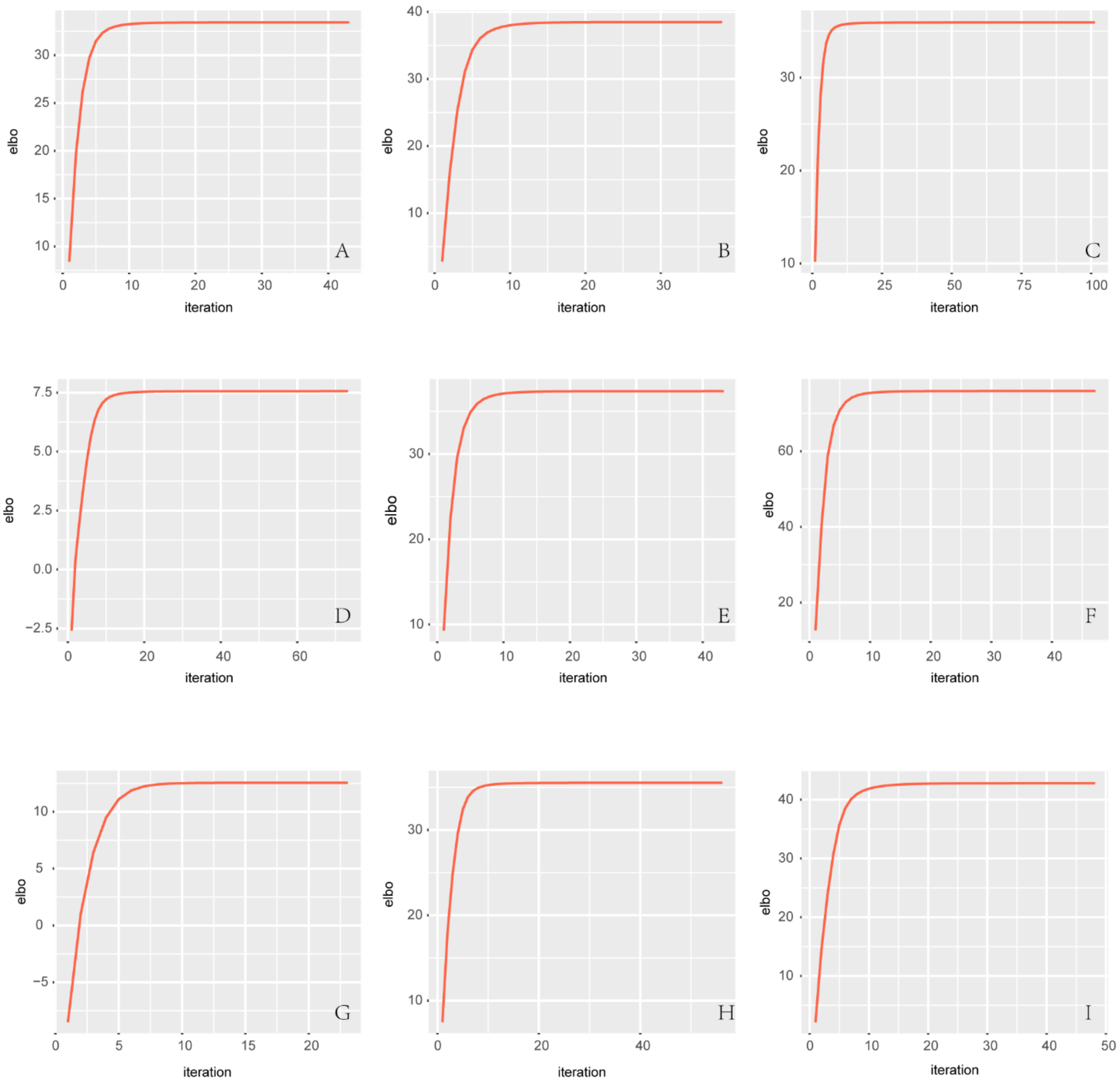


**Supplementary Figure 4** plot of evidence lower bound

A represents the causal relationship between Dorea and JIA;B represents the causal relationship between Rikenellaceae and JIA;C represents the causal relationship between Eubacteriumrectale and JIA;D represents the causal relationship between Catenibacterium and JIA; E represents the causal relationship between Ruminococcus and JIA; F represents the causal relationship between 1-stearoyl-2-oleoyl and JIA;G represents the causal relationship between Furaneol sulfate and JIA; H represents the causal relationship between Dorea and Furaneol sulfate; I represents the causal relationship between Rikenellaceae and 1-stearoyl-2-oleoyl.

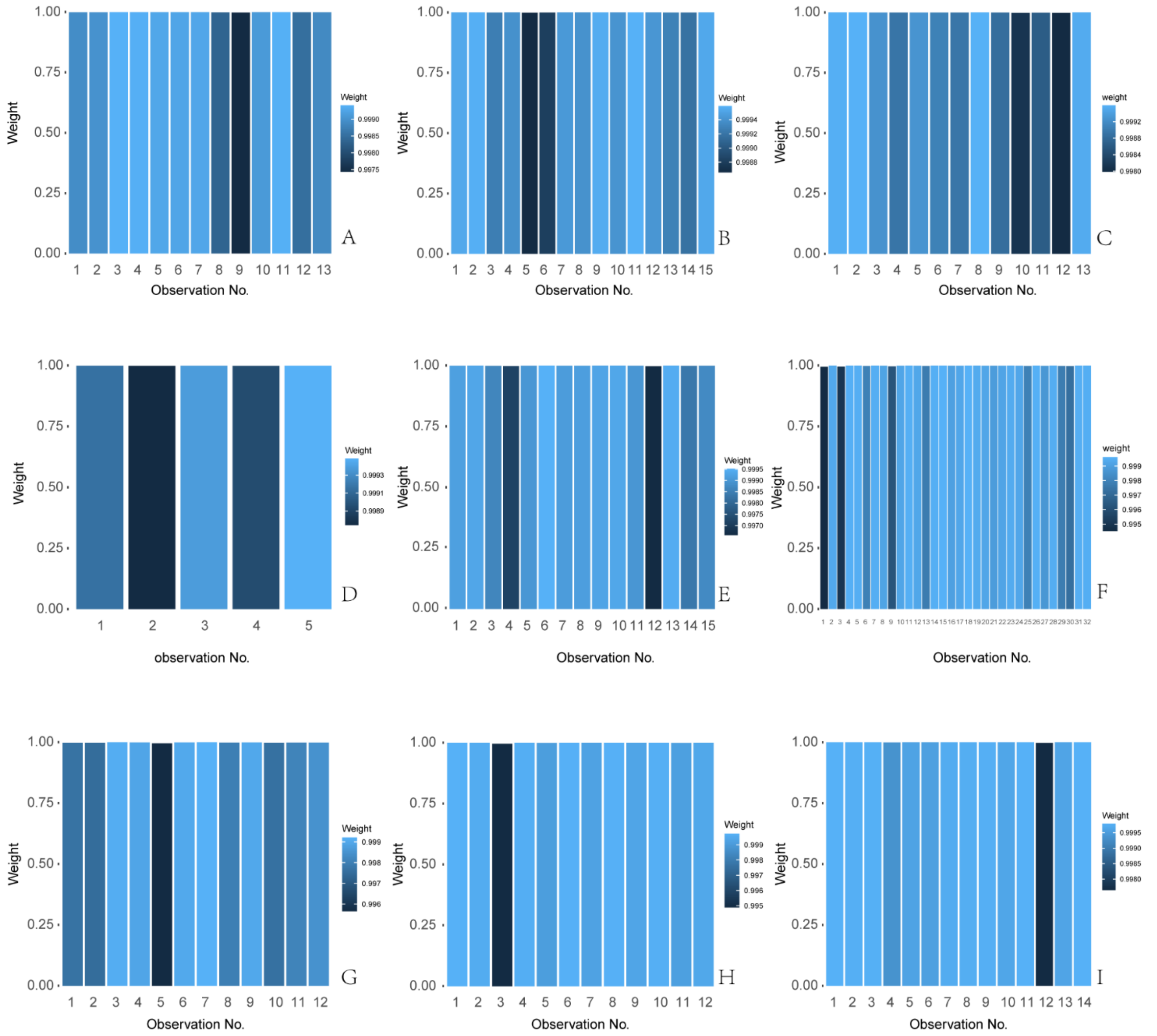


**Supplementary Figure 5** plot of posterior mean of weight of each observation

A represents the causal relationship between Dorea and JIA;B represents the causal relationship between Rikenellaceae and JIA;C represents the causal relationship between Eubacteriumrectale and JIA;D represents the causal relationship between Catenibacterium and JIA; E represents the causal relationship between Ruminococcus and JIA; F represents the causal relationship between 1-stearoyl-2-oleoyl and JIA;G represents the causal relationship between Furaneol sulfate and JIA; H represents the causal relationship between Dorea and Furaneol sulfate; I represents the causal relationship between Rikenellaceae and 1-stearoyl-2-oleoyl.

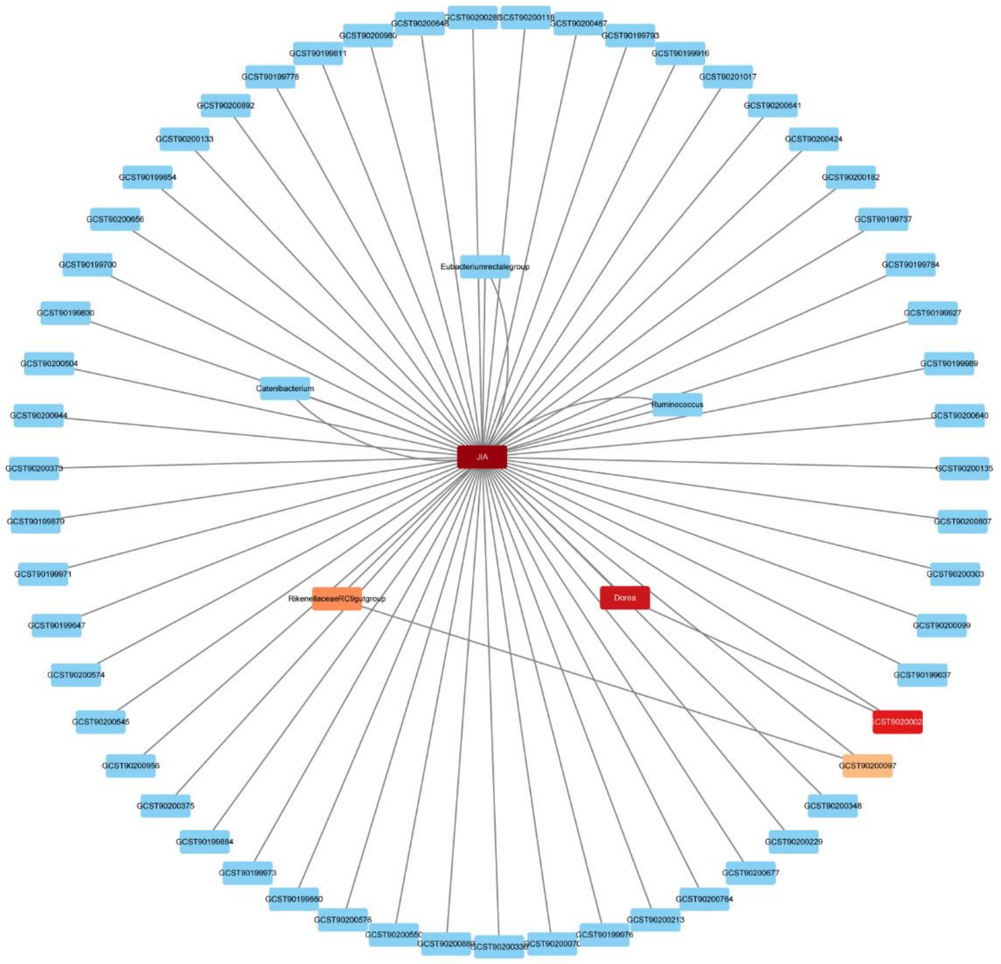


**Supplementary Figure 6** network of gut microbiota, plasma metabolites and juvenile idiopathic arthritis(JIA)
